# Reimagining Relationships: Multispecies Justice as a Frame for the COVID-19 Pandemic

**DOI:** 10.1007/s11673-023-10280-5

**Published:** 2023-08-25

**Authors:** Danielle Celermajer, Philip McKibbin

**Affiliations:** Sydney Environment Institute, Discipline of Sociology and Criminology, University of Sydney, Room 350 Social Sciences, A02, University of Sydney, Sydney, NSW 2006 Australia

**Keywords:** Relationality, Entanglement, Polycrisis, Indigenous worldviews, Ecofeminism, Multispecies justice, COVID-19

## Abstract

COVID-19 catalyzed a renewed focus on the interconnected nature of human health. Together with the climate crisis, it highlighted not only intra-human connections but the entanglement of human health with the health of non-human animals, plants, and ecological systems more broadly. In this article, we challenge the persistent notion that humans are ontologically distinct from the rest of nature and the ethics that flow from this understanding. Imposing this privileged view of humans has devastating consequences for beings other than humans and for humans and impedes effective responses to crises. We situate the COVID-19 pandemic within the “polycrisis,” and argue that one component of addressing multidimensional crises must involve fully embracing a relational ontology and ethics. We discuss two approaches to relationality, one articulated by ecofeminists and the second inhering in an Indigenous Māori worldview. Two dominant approaches, One Health and Planetary Health, purport to take account of relational ontologies in their approaches to health, but, we argue, persist in casting the more-than-human world in an instrumental role to secure human health. We suggest that Multispecies Justice, which draws on ecofeminist and Indigenous approaches, affords a fully relational approach to health and well-being. We explore the implications of relationality, and suggest fresh ways of understanding humans’ connections with the more-than-human world.

## Introduction

The globalization thesis—that people and institutions across the globe have become increasingly connected over the last fifty-odd years—has by now become banal. Nevertheless, the global pandemic that began in early 2020, and persists at the time of writing in late 2022, afforded many people an immediate experience of how such connectedness now plays out in the form of globally shared vulnerability, traversing scales from the global to the most intimate and everyday aspects of individuals’ and communities’ lives. The zoonotic origins of the virus were exposed early on and the experience of this pandemic brought into the public sphere debates that have been happening across a range of disciplines about the embeddedness of human well-being in the more-than-human world. While the global response has emphasized vaccine development and a range of public policy measures aimed at curbing disease transmission, there has also been broad recognition of the link between the development of the disease in humans and humans’ deleterious impacts on the more-than-human world, including ecosystem degradation and disturbance, habitat destruction, globalization of the live animal trade, intensive animal farming, and the rapid climate change forced by contemporary forms of human life (Barouki, et al. 2021; Caminade, McIntyre, and Jones 2019; Carlson, et al. 2022; Halabowski and Rzymski 2021). Correlatively, the differential experience that individuals and communities have had of the disease has been influenced by a range of social or health factors, such as comorbidities and overcrowding, themselves rooted in a similar range of ecological pathologies (Han, et al. 2022; Millet, et al. 2020).

In this regard, the impacts of COVID-19 belong to a larger set of interlocking trends of massive disruption—in ecological systems, in biodiversity, in politics, in the economy, in food systems, and elsewhere, which is coming to be known as the polycrisis (Homer-Dixon et al. 2021; Homer-Dixon and Rockström 2022) or even more direly, the age of collapse (Servigne and Stevens 2020). While the analysis of the polycrisis is in its early stages, naming it has already led to calls to more comprehensively document the

… complex and dynamic networks of multiple, synergistic causes and feedback loops … the highly nonlinear cause-effect relationships … [and] causal processes that cross boundaries of administrative and political units, social sectors, and scientific disciplines and that operate on multiple time scales across natural, social, and technological systems. (Homer-Dixon et al. 2021, 4)

By analysing the COVID-19 pandemic as one manifestation of the polycrisis, this article seeks to contribute to this larger project by arguing that neither the pandemic nor the polycrisis can be understood, nor can a path out of them be created, without attending to the ontology and ethics of human exceptionalism that underpin them. So long as any problem analysis remains steeped in an ontology that casts the being of human individuals or even human populations as fundamentally separate from the more-than-human world, and an ethics where humans are assumed to be of superior moral value, and all others means to the pursuit of human ends (Chakrabarty 2021; Kant 1988 [1786]), the complex of pathological factors will remain locked in place. Transforming the foundational ontological and ethical frameworks that underpin the current pathological situation and assuming an ontology and ethics of radical relationality will not be sufficient to overcome it, but both moves are necessary.

In this, there is bad as well as good news. The bad news is that ideological commitments to human exceptionalism, ontological and moral individualism, and the extractive relationship with the more-than-human that follows have been intrinsic to the forms of human life and social, economic, and political arrangements, in particular colonialism and capitalism, that are both cause and condition of many aspects of the polycrisis (Preston 2017; Grosfoguel 2019; Nygren, Kröger, and Gills 2022). The good news is that there is no shortage of alternatives where relationality and the rejection of human exceptionalist moral hierarchies are foundational, and they exist not only as abstract frames of meaning but as persistent forms of life (Celermajer and Winter 2022).

In this short article, we briefly canvass two such alternatives, which we call “foundational,” namely ecofeminism and a Māori worldview, and one recently emergent approach that significantly rests on the former two and is directly relevant to the COVID-19 pandemic—Multispecies Justice. In this admittedly brief disucssion, we hope to achieve three things: first, to demonstrate the availability of alternative ontological and ethical frameworks, both within western thought (ecofeminism) and outside it (Māori worldviews); second to draw out from them some of the principal features of relational ontologies and ethics; and third, to offer those trying to think through the crisis some intellectual resources capable of nurturing resistance and transformation. In presenting Multispecies Justice as an emergent ethical frame for approaching the pathology of the polycrisis out of which the pandemic emerged, our focus is more specifically on how it departs from two health approaches that would seem to, but, we argue, do not sufficiently, address the complex of human exceptionalism or embrace ontological relationality: One Health and Planetary Health.

## Ecofeminist Perspectives and Relationality

Ecofeminism is often associated with Carolyn Merchant’s (1980) articulation of the shared roots and interlocking structures of the oppression of, and violence against women and “nature” (Warren 1990; Gaard 1993). Critically, in doing so, ecofeminists were not, as they have been accused of, making essentialist claims about a natural affinity between “women” and “nature,” but on the contrary, pointing to the ways in which particular forms of science and political economies, that is rationalism and capitalism, have combined to create the discursive frame and institutions that linked, operationalized, and normalized the association and oppression of women and nature.^1^ For our purposes though, the part of this institutional and discursive complex that is particularly relevant is its construction of a dualistic framework within which the idea that human progress (both ethically and in terms of human flourishing) is to be achieved. This entails several (non-sequential but rather linked) moves. First humans must assume as their fundamental identity what is posited as essentially and uniquely human (understood evaluatively and not merely descriptively), that is, transcendent reason, understood as radically separate from the “what” that belongs to the determined world of “nature.” Second, it is morally right for the rejected and expelled “nature” to be used as means towards the ultimate moral ends, the enhancement of the transcendent (human) self. This process rests on an ontological logic, the creation *of* a “supposedly sharp separation, cleavage, or discontinuity between all humans and the nonhuman world, and the similar cleavage within the human self” (Plumwood 1991, 6).

Thus, for example, just over thirty years ago, the ecofeminist philosopher Val Plumwood, characterized the “standard Western view of the relation of the self to the nonhuman” as one in which the latter “can be used as a means to the self-contained ends of human beings. Pieces of land are real estate, readily interchangeable as equivalent means to the end of human satisfaction; no place is more than ‘a stage along life’s way, a launching pad for higher flights and wider orbits than your own’ (Berman 1982, 327)” (Plumwood 1991, 20–21). “Nature,” and all those humans, who by virtue of their gender or race or other attributes, are classified as belonging on the side of nature (Jackson 2020; Kim 2015; Weheliye 2014), are then posited as “dead matter,” “terra nullius, resources empty of their own purpose and meanings” and available for use without need for justification by those capable of transcendence (Plumwood 1993, 4, 45ff).

Critically, the alternative ecofeminism articulates is neither a reversal, although Plumwood does argue for resituating “humans in ecological terms and non-humans in ethical terms” (Plumwood 2005, 8–9), nor the dissolution of all boundaries, as she diagnoses to be the case for deep ecology.^2^ Extending intrinsic value from humans to nature, she argues

… neglects a key aspect of the overall problem that is concerned with the definition of the human self as separate from nature, the connection between this and the instrumental view of nature, and broader political aspects of the critique of instrumentalism. (Plumwood 1991, 6)

What is required then, is a reconceptualization of “the human” and of “the self” as understood and performed in hegemonic western forms of life.

Reconceptualizing the human involves reincorporating into the human as both essential and valuable those qualities that have been “split off, denied or construed as alien,” and correlatively, rejecting the mechanistic view of nature, henceforth defined as the repository of what had been split off and denied. Recasting the self, in turn, involves a rejection of the idea of the self as radically separate, where all others are at best of instrumental value in pursuit of the self’s ends. The alternative though, is not to aspire for a self indistinguishable from all else, or expanded to encompass all beings, for this would mean forsaking all possibility of distinction, autonomy and difference, and hence of recognition of the *otherness* of others. In other words, it is not *difference* that is the problem, but difference refracted through hierarchical dualisms. Moreover, it is not the existence of self and other that is the problem, but the external and oppositional relationship within which they are cast. What is required is a more radical recasting of the self as relational, where, a Karen Warren puts it, “Relationships are not something extrinsic to who we are, not an ‘add on’ feature of human nature; they play an essential role in shaping what it is to be human” (Warren 1990, 143). Thus, whereas for Kant and the tradition that follows him (Chakrabarty 2021; Celermajer and Winter 2022), it is only by virtue of recognizing other humans as also radically autonomous ends in themselves that it is rational to accord them moral worth, here “respect for others … is an expression of self in relationship” (Plumwood 1991, 20). For the relational self, others’ good, including what is good for “nature” or other animals is not accidently, but intrinsically part of the good of the self, not because they are merged but because they are related all the way down.

The ecofeminists writing in the 1980s did not, of course, have the pandemic in mind, but their call for recasting the human and the self has clear applications to this context. In light of what we now know about what has brought us to this moment, and specifically about the link between the ecological destruction that is an inescapable consequence of contemporary forms of human life under extractive capitalism on the one hand, and the production of the conditions for pandemics, ecofeminists’ philosophical approach is highly apt. If one follows their analysis of the pathologies generated by the refusal of relationality that characterizes western rationalism and capitalism, what follows is that attending to the good of “nature” is not a luxury, a goal to which we humans might aspire, once “our” interests have been met, in an imagined Maslowian hierarchy of needs, but intrinsic to human good.

As Plumwood already acknowledged, however, the grip that a dualistic ontology of radical discontinuity has on the West is by no means universal and “many other cultures … stress what connects us to nature as genuinely human virtues, which emphasize continuity and not dissimilarity” (Plumwood 1991, 10). Plumwood had learned this from First Nations people in Australia, but it is to Māori ontologies and ethics that we now turn.

## Māori Perspectives on Relationality

Indigenous worldviews can assist all of us in thinking through relationality and in moving past the profound ontological and ethical hierarchies within which hegemonic Western thought remains entrenched. We turn to te ao Māori (the Māori world) as an example, in part because one of the authors of this paper is Māori; McKibbin is related, through ancestry, to Kāi Tahu, an iwi (tribe) of Te Waipounamu (the South Island) of Aotearoa New Zealand. We believe a focus on whakapapa (genealogy) and three prominent Māori values can helpfully inform our exploration of relationality. These values are whanaungatanga (relationship), tiakitanga (caring), and hauora (well-being). These values are widely shared throughout te ao Māori; however, the ways in which they are interpreted, and put into practice, vary significantly across iwi (tribes), hapū (sub-tribes), and whānau (families).

A Māori worldview understands humans as related, through whakapapa (genealogy), to the rest of the natural world. As Georgina Tuari Stewart notes,

Whakapapa is a master concept of the Māori worldview and key to understanding Indigenous Māori views of the natural and social worlds, and guiding right ethical relationships between people, and between humans and other living and non-living things. The nature narratives of the Māori creation story are a model for how the concept of whakapapa works for organising arrays of complex information. (Stewart 2021, 85)

The idea that all life is connected through genealogy is analogous, in some ways, to evolutionary theory, in that it connects us back to previous generations, and acknowledges the way environments structure interactions between generative forces. However, on a Māori worldview, much of that which is considered “non-living” by Western science is not only understood as having its own mauri, or “life force,” but is also personified, and perceived relationally. Moreover, as Christine Winter observes, in a Māori worldview, time does not simply move forward, along a linear and one directional path, but takes a spiral form (Winter 2020). 

Hirini Moko Mead writes, “Whanaungatanga embraces whakapapa and focuses upon relationships” (Mead 2016, 31). Although the concept of whanaungatanga is typically understood primarily in terms of human relationship, it is also informed by the ontological recognition that all the dimensions of the natural world are connected through genealogy. Understood in this way, whanaungatanga animates those connections and it suggests responsibilities between us. As well as recognizing that humans are “of” the more-than-human world, it construes us in ongoing, reciprocal relationship with it.

Another Māori value that might inform how we understand relationality is tiakitanga (caring). Tiakitanga encompasses the obligations of care that humans have to the more-than-human world, and the integral role we play in its well-being. It is closely related to—and can even be understood as a component of—kaitiakitanga (environmental management), which emphasizes the role of human stewardship, via the prefix “kai-” (Kawharu 2000). (Although we believe both concepts are instructive, we deliberately focus on tiakitanga over kaitiakitanga because the former emphasizes caring itself rather than privileging human givers of care.) Significantly, the notion of tiakitanga encourages us to relate to the more-than-human world in ways that are humble, nurturing, and respectful.

Moreover, Māori understandings of hauora (well-being) suggest that human health can be conceived in ways that affirm not only intra-human relationships but relationships with the more-than-human world. Māori models of well-being are typically pluralistic and situate the individual not only within the collective, but within the wider environment.

For example, Te Whare Tapa Whā, a model developed by Mason Durie, utilizes the image of a building, the four walls of which—“taha wairua (the spiritual side), taha hinengaro (thoughts and feelings), taha tinana (the physical side), [and] taha whānau (family)” (Durie 1998, 69)—comprise a person’s well-being. This model has gained wide acceptance in te ao Māori (the Māori world). As Durie explains, “Though often described as a traditional Māori approach to health, more correctly it was a view of health which accorded with contemporary Māori thinking. Its ready acceptance by Māori was to some extent proof of that” (Durie 1998, 68–69). Each of these four “sides” emphasizes relationality. The taha whānau (the family side) “acknowledges the relevance of the extended family to health” (Durie 1998, 72), acknowledging the family as the primary source of support, and understanding interdependency as a strength. Relatedly, the taha wairua (the spiritual side), which “is generally felt by Māori to be the most essential requirement” entails, in part, that one is “able to understand the links between the human situation and the environment” (Durie 1998, 70), and acknowledges the inherent value of the natural world.

Similarly, Rangimarie Turuki Pere’s model, Te Wheke–which reaches “into the past, present and future of the ancient teachings of Hawaiki” (the traditional Māori homeland) (Pere 1991, 3), and which has also gained a wide level of support—also emphasizes the relational nature of well-being. This model encourages us to imagine well-being as the body (i.e. the head, suckers, and tentacles) of an octopus. Among its emphases are “whanaungatanga” (relationship, or “kinship ties”), which holds that “everything across the universe is inter-related” (Pere 1991, 26), and whenua (which means both land and placenta in te reo Māori). “The placenta embracing and cherishing the child in the womb is called whenua. [T]he land which is also called whenua offers the same feeling of warmth, security, nourishment and sustenance, a feeling of belonging” (Pere 1991, 22). As Pere explains, “The tentacles can also be intertwined so that there is a mergence, with no clear-cut boundaries. The dimensions need to be understood in relation to each other, and within the context of the whole” (Pere 1991, 3).

When the health of one is understood as contingent upon right relationship with others, those relationships are understood as entailing obligations of justice. These concepts, which are fundamental to Māori ways of being, suggest some ways that relationality might structure our understanding of our place in the world. Such values are not always put perfectly into practice; however, as Māori Marsden, who wrote during the twentieth century, and whose work was posthumously collected, argues,

Until we relearn the lesson that man [sic] is an integral part of the natural order and that he has obligations not only to society but also to his environment so long will he abuse the earth. To realize that he is a child of the Earth will help him in working to restore and maintain the harmony and balance which successive generations of humankind have arrogantly disturbed (Marsden 2003, 69).

We mention these Māori concepts—whakapapa, whanaungatanga, tiakitanga, and hauora—not because we believe that Indigenous worldviews hold all the answers to our collective problems, nor because we think that the notions they carry, or the metaphysics that inform them, must always be accepted uncritically. Rather, we are convinced that by attending to them, and a diversity of perspectives, we will better understand what it means to relate well, and that this could helpfully inform our efforts at elaborating multispecies justice for te ao hurihuri (our changing world).

The world we have known all our lives is unravelling. As we seek to weave new ways of being and doing out of knowledges old and new, we will fare better if we work together and learn from all of our experiences, including Indigenous worldviews.

## A Relational Framework for Addressing Health

In the field of health, the idea of relationality already has significant recognition. That human health is connected to the health of what we might broadly call the more-than-human—that is, animals other than humans (hereafter other animals), plants and fungi, and ecological systems—has already led to the development of alternative approaches to the traditional narrow focus on health amongst human populations alone. The Planetary Health model, which “emerged from the environmental and holistic health movements of the 1970–1980s” (Logan et al. 2020, 8), recognizes that human health and the health of the planet are inextricably linked (Pongsiri, et al. 2019; Salk 2019; Redvers, et al. 2020). As Pongsiri et al. explain,

Planetary health sets the ambitious task of understanding the dynamic and systemic relationships between global environmental changes, their effects on natural systems, and how changes to natural systems affect human health and wellbeing at multiple scales: global (eg, climate), regional (eg, transboundary fire emissions), and local (eg, persistent organic pollutants). (Pongsiri et al. 2019, E402)

This approach has helped frame efforts at protecting the environment. For example, it motivated the UNFCC’s “Momentum for Change” project (United Nations 2016).

The One Health model has a similar, albeit slightly broader focus, recognizing connections between human, animal, and environmental health (de Macedo Couto and Brandespim 2020). This model “initially emerged from the integrated study of zoonoses” (de Macedo Couto and Brandespim 2020, 82), and in light of the COVID-19 pandemic—caused by a virus which is believed to have originated in non-human animals before spreading to humans (Ye et al. 2020)—has been embraced by the World Health Organization (World Health Organization 2021). Like the Planetary Health model, it affirms the importance of environmental protection efforts, and it has prompted a renewed focus on the ways in which animal health bears on human health.

These paradigms represent important shifts in how human health is conceived, embedding it in systems of interdependence and demanding that researchers, practitioners, and policymakers pay attention to the health of the more-than-human. However, against the background of the argument we have made, the critical question is whether they authentically adopt a relational ontology and ethics. Given the breadth of both approaches and range of programmes that have developed under their banners, we do not presume to comprehensively or accurately answer that question, but rather posit it as one that needs to be asked and suggest that there are reasons to be sceptical about the depth of their relational ontology.

Concerns have been raised that even as they adopt a systems approach and expand their gaze beyond the human, neither approach has authentically moved beyond a value system where humans and humans alone have ultimate moral value, and that, although included in their purview, the health of the more-than-human is ultimately relegated to instrumental value. As Irus Braverman notes in the introduction to the recent critical collection, *More-than-One Health*, Planetary Health “maintains a focus on human health, only from a global perspective” (Braverman 2022, 4). Maneesha Deckha makes a similar observation of the One Health paradigm, arguing that because it continues to construe animals as resources, rather than as subjects with rights, “the mission of the OHI [One Health Initiative] and similar initiatives remains compromised and the attainment of their objectives elusive” (Deckha 2023, 166).

In other words, although both the Planetary Health and One Health models do endeavour to understand connections between human health and the more-than-human world, they do so primarily—and, usually, *only*—for the sake of human health. This charge seems to gain support from a comparison of the response to the recent human pandemic and the response to a threatened animal pandemic. If we focus on Australia as a case study, when it came to COVID-19, even the economically conservative neo-liberal government made massive policy and economic shifts to protect human well-being and human welfare, including rapidly (albeit temporariliy) expanding the welfare state. During this same period, where Australia contemplated an outbreak of Foot and Mouth Disease, already present in cows in Indonesia, the policy envisaged a combination of vaccination and mass slaughter (Australian Ministers Forum 2014). And while the policy mentions the welfare of animals in relation to slaughter and management, the discussion of impacts is limited to economic and social costs for human individuals, families, and communities.

This is hardly surprising, given that in almost all contemporary societies, and amongst the organizations that have been most involved in developing these paradigms, anthropocentrism and a hierarchical value system, in which humans are unquestionably at the top, remain hegemonic. On the one hand, one might argue that this does not matter, so long as there is a focus on the health of others. We would suggest however that this slippage between an apparent transformation in who or what is included as an object of study or treatment, and the actual stasis with respect to the more fundamental ontological and ethical questions, is critical from the point of view of robustly addressing the crises of health and the larger polycrisis within which it is embedded. Insofar as the new approaches fall short of this more foundational transformation, they reinscribe the very logics of human exceptionalism and extractivism that have led to the destruction of environments and the destruction of animal habitats and lives that are at the root of the current heath crisis.

By contrast, we suggest that Multispecies Justice offers a framework for rethinking health in a way that comprehensively embraces a relational ontology and ethics. This is not least because Multispecies Justice arose as a distinct framework in part in response to the limits of liberal theories of justice, limits that themselves emerged from liberalism’s anti-relationality and explicitly drew on relational approaches, including prominently ecofeminism and Indigenous worldviews (Celermajer et al. 2021; Celermajer, Schlosberg, Wadiwel and Winter 2023; Celermajer and Chang forthcoming). Māori perspectives in particular have been integral to early theorizing around Multispecies Justice and can be seen in the contributions made by Makere Stewart-Harawira and Christine Winter (Celermajer et al. 2021; Tschakert et al. 2021, Winter 2022).

As an emerging field, Multispecies Justice does not have a credo and is not strong theory, in the sense Gibson-Graham uses it as being a powerful discourse “that organize[s] events into understand-able and seemingly predictable trajectories” (Gibson-Graham 2014, S148). Nevertheless, we identify three commitments that are foundational to Multispecies Justice that qualify it as a framework that would support the type of transformation in thinking about health required to address emerging pandemics, understood as part of the polycrisis. First, and continuous with the argument made throughout this article, Multispecies Justice adopts a relational ontology and ethics rather than an extensionist approach. Second, by adopting the framework of justice, it insists that attention to the good of the more-than-human is not an additional or defeasible consideration but a requirement of the fundamental institutions of society. Third, while the name draws attention to the inclusion of the more-than-human within the reach of justice, it is attentive to intra-human injustices and their structural relationships with injustice committed against the more-than-human world, including the structures of colonialism, capitalism, and forms of domination that structure systematic inequality. Below, we briefly describe each.

First, while Multispecies Justice as a field has certainly been influenced by a range of ecological and animal justice movements, unlike the rights-based or utilitarian approaches that have often dominated the field of animal ethics in particular, it does not take an extensionist approach. Certainly one of the justifications for Multispecies Justice has been the dissolution of many of the claims that have traditionally been used to justify absolute human difference, but the criterion for the recognition of the moral considerability of beings other than humans is not their possession of the characteristics that have grounded moral considerability for humans, such as the capacity to suffer or to reason. Rather, grounded as it is in a relational ontology, its demand for a capacious approach to justice or ethics is based on the idea that the good of one class of beings (humans or some humans) cannot be thought or achieved abstracted from the broader more-than-human world that constitutes humans themselves and their good. Importantly, this is not a way of saying that the environment or other animals have high instrumental value for humans and thus ought to be protected. As Plumwood argued, the relationship is not accidental or extrinsic, but fundamental.

Second, insofar as it is a theory of justice, Multispecies Justice is making claims about what is required from societies’ basic institutions, where institutions are understood as “sets of rules and norms, both explicit and implicit, that work to structure social interactions, and which give rise to normative expectations and stable patterns of behaviour” (Celermajer, et al. 2019, 4). To say that a claim is one of justice is to say that it cannot be dismissed because it is inconvenient, preferences change, or majorities discount it. This does not mean all claims made in the name of justice are absolute and must be met in some absolute sense. It rather means that a failure to honour them must be justified in a way that is neither arbitrary nor based on discounting the identity of the claimants, especially on the basis of assumed distinctions in their value. Asserting that the more-than-human world, animals, and the environment are subjects of justice takes attending to their claims out of the realm of generosity and demands that they be subject to the types of institutional procedures put in place to seriously respond to the justice claims of humans (Eckersley 2011). In this regard, the existing laws in almost all jurisdictions, domestically and internationally, that purportedly protect the interests of the environment and other animals are not laws of justice, because those interests are almost always rendered defeasible and automatically discounted vis a vis human interests.

Third, Multispecies Justice brings a structural and historical analysis to the systematic injustice that characterises intra-human relations and relations between hegemonic contemporary forms of human life and the more-than-human. In this regard, the dynamics of capitalism, colonialism, and their intrinsic relations with racism and gender injustice are not supplementary considerations but internal to Multispecies Justice (Celermajer, et al. 2021). Connecting this multidimensional analysis with the COVID-19 pandemic, the link between violence against the environment and other animals, and the highly uneven impacts of access to treatment and social and economic suffering as a result of the pandemic noted at the outset of this article is not accidental, but a product of the structures that produce injustice for the more-than-human world and certain humans. Indeed, this brings us right back to the ecofeminist analysis of the dualistic and hierarchical structures that organise rationalism and capitalism, relegating certain classes of beings to the status of resource to be extracted for the good of others. It also brings us back to the decolonial analysis that refuses the assertion that there is only one way to properly organize human society, only one correct understanding of the human and the more-than-human, and that the different ways diverse humans groups organize or understand their worlds are hierarchically ordered.

## Conclusion

If we are to address the polycrisis, of which the COVID-19 pandemic is one manifestation, we must entrench in our approaches a relational ontology and ethics. This requires overcoming the ideological commitment to human exceptionalism which, with colonialism and capitalism, came to dominate contemporary social, economic, and political arrangements, as well as the individualistic and extractivist logics that extend from it. As demonstrated in ecofeminist approaches and Indigenous worldviews, we must work to realize ways of relating that affirm our interconnectedness. Multispecies Justice, with its commitment to relationality, encourages us to re-think health and well-being, to reconsider who and what matters, and to explore the ways in which we are all related.

## Footnotes

1 That ecofeminism includes a strong critique of capitalism is often overlooked but clear in Plumwood. For example, Plumwood is critical of the failure to account “for connections with a number of other critiques, not just feminism, for example, but also socialism, especially in the forms that mount a critique of rationalism and of modernity. The failure to observe such connections is the result of an inadequate historical analysis and understanding of the way in which the inferiorization of both women and nature is grounded in rationalism, and the connections of both to the inferiorizing of the body, hierarchical concepts of labor, and disembedded and individualist accounts of the self” (Plumwood 1991, 22).

2 As Plumwood herself notes and as we develop below, this resituating is not required for all humans or human forms of life.
